# Molecular and Functional Characterization of Elicitor PeBC1 Extracted from *Botrytis cinerea* Involved in the Induction of Resistance against Green Peach Aphid (*Myzus persicae*) in Common Beans (*Phaseolus vulgaris* L.)

**DOI:** 10.3390/insects10020035

**Published:** 2019-01-24

**Authors:** Abdul Basit, Abdul Hanan, Talha Nazir, Muhammad Zeeshan Majeed, Dewen Qiu

**Affiliations:** 1 Key Laboratory of Integrated Pest Management in Crops, Institute of Plant Protection, Chinese Academy of Agricultural Sciences, Beijing 100081, China; malikbasituaf@gmail.com (A.B.); hunnyuos@gmail.com (A.H.); talha23december@gmail.com (T.N.); 2 Department of Entomology, College of Agriculture, University of Sargodha, Sargodha 40100, Pakistan; zeeshan.majeed@uos.edu.pk

**Keywords:** elicitor proteins, PeBC1, *Myzus persicae*, life-history traits, jasmonic acid pathway, salicylic acid pathway

## Abstract

Elicitors are biofactors that induce resistance in plants against different insect pests. This in vitro study evaluated the impact of a novel elicitor protein PeBC1, extracted from a necrotrophic fungus *Botrytis cinerea*, on the development and fecundity parameters of green peach aphid (*Myzus persicae*) on common beans (*Phaseolus vulgaris* L.). Three different concentrations of PeBC1 elicitor (i.e., 33.56, 25.43, 19.33 µg mL^−1^) were applied at three different temperature regimes (i.e., 18, 21, and 25 °C). Elicitor treatments were applied topically on the bean plants at 3-leaf stage and newly emerged (0–6 h old) apterous adult aphids were exposed to these treated leaves. In addition to the biological parameters of aphids, the relative expression levels of key genes associated with jasmonic acid (JA) and salicylic acid (SA) plant defense pathways were also determined through RT-qPCR. Results of bioassays revealed that the application of PeBC1 elicitor protein exhibited pronounced and significant (*p* < 0.05) sub-lethal effects on green peach aphids. The fecundity was reduced and the nymphal development time was prolonged by different concentrations of PeBC1 elicitor and temperature regimes. Gene expression studies showed that the exogenous application of PeBC1 induced a significant upregulation of the expression levels of JA and SA pathway-associated genes in bean plants. As compared to control, elicitor-treated plants exhibited an induced resistance against aphids. Our findings suggest the potential use of PeBC1 elicitor protein in future bio-intensive management strategies against sap-sucking insect pests such as green peach aphids.

## 1. Introduction

Elicitors are biofactors or chemicals used by plants under attack as signal molecules to induce systemic acquired resistance against pathogens or herbivores by activating different defense pathways [1,2]. Elicitors are categorized as microbe-associated molecular patterns (MAMPs), which are usually derived from the microbes and herbivore-associated molecular pattern (HAMPs) produced by herbivorous insect pests. HAMPs have mostly been characterized from lepidopterous, dipterous, and orthopterous pests [1]. Volicitin, for instance, was the first herbivore-induced elicitor characterized from beet armyworm (*Spodoptera exigua*) [3]. Similarly, different types of elicitor proteins (MAMPs) have been identified from fungal (e.g., Pep-13 and *endo*-β-1,4-xylanases from *Phytophthora* and *Trichoderma*, respectively) and bacterial (e.g., *flg22* from bacterial flagella) pathogens [4,5,6]. These elicitors play an important role in crop protection, as they can induce plant resistance to pests, reduce pest fitness, and deter their feeding.

Elicitors usually include proteins, glycoproteins, and lipids that induce resistance in plants against pathogens and herbivore pests [2,7,8] through the activation of various response mechanisms in plants, such as signaling pathways, hypersensitive response (HR), and reactive oxygen and nitrogen species (ROS and RNS, respectively) responses [9]. Commonly occurring processes such as protein phosphorylation or activation of plasma membrane proteins produce various direct as well as indirect signaling molecules, such as ROS and nitric oxide (NO), that regulate metabolic and transcriptional changes through physiological response [2]. 

Jasmonic acid (JA) and salicylic acid (SA) are two of the most important signaling molecules that enhance the plant defense responses [10]. According to the current theory, plant response to herbivory and necrotrophic pathogen infestations triggers the JA and SA defense pathways [10]. Similarly, some elicitors and eliciting components can act as resistant protein- and nucleotide-binding factors in plants that lead to resistance against insect pests such as aphids [11,12].

Entomopathogenic fungi are of great importance in the biological control of insect pests due to their low mammalian toxicity and high host specificity [13]. Moreover, these fungi have the ability to develop themselves like entophytes within different plant portions [14,15]. Furthermore, entomopathogenic fungi produce systemic resistance in plants against biotic stresses such as phytoparasites, pathogens, and nematodes [16], enhance plant growth [17], increase yield [18], and improve nutrition of the plants [19] and root growth [20,21]. In addition, these fungi help the plants to mitigate abiotic stresses such as drought [22], iron chlorosis [23], and salinity stress [24]. These new ecological functions of fungi provide potential benefits to plant health and provide a new perspective to the development of novel plant protection strategies [23]. Similarly, some elicitor proteins have recently been isolated from necrotrophic and biotrophic fungal pathogens manifesting induced resistance against pathogens and herbivores. For instance, the elicitor PeBC1, cloned from a necrotrophic fungus *Botrytis cinerea*, induced resistance against diseases in *Arabidopsis thaliana* [25]. 

This study was aimed to investigate the activity and molecular mechanism of the *B. cinerea*-derived elicitor protein PeBC1 in the induction of plant resistance in common beans (*Phaseolus vulgaris*) against green peach aphids. As the activity of plant defense signaling pathways mediated by elicitor proteins may be modulated by different environmental factors, such as temperature [26], the response of aphids to PeBC1 elicitor was also assessed under different temperature regimes. 

## 2. Materials and Methods

### 2.1. Insects and Plant Culture

Common bean (*P. vulgaris*) plants were grown in plastic pots in sterile soil mix. Plants were grown in a greenhouse under natural light with day and night temperature varying from 21 to 33 °C. Effects of treatments on the plants were observed by measuring the appropriateness of foliage from elicitor-treated and control plants for aphid growth in short-term feeding bioassays. A colony of green peach aphid, *M. persicae*, was maintained on bean plants in isolation cages in a growth chamber. The colony was established for three months prior to the onset of experiments to ensure that aphids were suitably adapted to the chemistry of the bean plants. Aphids were reared at 21 ± 2 °C temperature and 65% to 75% relative humidity (RH).

### 2.2. Protein Purification

Elicitor protein PeBC1 was extracted from the colony of *B. cinerea* cultured in 1 L of LB medium at 200 rpm for 12 h at 37 °C. The pellets were collected and cells were re-suspended and broken by sonication. After the centrifugation of the solution at 12,000 rpm for 15 min, the supernatant was collected and filtered through a filter paper (size 0.22 µm). Further purification of the elicitor protein was carried out by affinity chromatography with a HisTrap^®^ HP column (GX-11860073, GE Healthcare, Munich, Germany) using different loading buffers (A, B, C, and D). Buffer A (50 mM Tris-HCl, pH 8.0) simply washed other elicitors from the column, while buffer B (50 mM Tris-HCl, 200 mM NaCl) was used to stabilize the column. Buffer C (50 mM Tris-HCl, 200 mM NaCl, and 20 mM imidazole, pH 8.0) and elusion buffer D (50 mM Tris-HCl, 200 mM NaCl, and 500 mM imidazole, pH 8.0) were used for elution of elicitor protein from the solution. Subsequently, elicitor was desalted using desalting tubes and centrifugation was done at 4500 rpm at 4 °C. The molecular mass of the extracted elicitor protein was determined by 12% SDS PAGE resolving gel and a protein marker was used to estimate the molecular mass of the purified recombinant elicitor.

### 2.3. Bioassays with Elicitor Protein PeBC1

Elicitor protein concentrations were measured in the stock solution using a Bradford assay. Three different concentrations of PeBC1 elicitor protein (i.e., 33.56, 25.43, 19.33 µg mL^−1^) were used along with the buffer (50 mM Tris-HCl, pH 8.0) as control to determine the effectiveness of this elicitor against aphids. Bioassays were started at 3-leaf stage of the bean plant. Elicitor treatments were applied with an aerosol spray bottle until the bean plants were thoroughly covered and began to drain. Approximately 2 to 3 mL of elicitor solution was applied per plant. Control plants were sprayed with buffer (50 mM Tris-HCl, pH 8.0) only. Plants were allowed to dry for 24 h and then 3 to 5 freshly molted (0–6-h-old) apterous adult aphids per leaf were released. The development time was recorded for each offspring of these released aphids from 1st instar until the adult emergence and the fecundity was calculated as the total number of offspring produced by these newly emerged adults by careful observations at regular intervals of 3 h throughout the duration of bioassay. Each treatment was replicated 10 times and bioassays were repeated three times independently at three different temperature regimes (i.e., at 18, 21, and 25 °C).

### 2.4. Isolation of RNA and cDNA Synthesis

For molecular characterization of elicitor-induced resistance against aphids, plants were treated with 33.56 µg mL^−1^ concentration of PeBC1 elicitor and aphids were allowed to feed on these treated and untreated (control) plants for the same time period as in the previous bioassay. Leaf samples were then collected from these treated and control plants and after treating with liquid nitrogen were stored at −80 °C until use. RNA was extracted from these leaf samples by using EasyPure^®^ Plant RNA Kit ER301-01 (TransGen Biotech, Beijing, China) following the manufacturer’s protocol. Quantity and quality of the extracted RNA were measured with NanoPhotometer^®^ (NP80 Touch, Implen Inc., Westlake Village, CA, USA). After this, RNA was reverse transcribed to cDNA by using TransScript^®^ First-Strand cDNA Synthesis SuperMix AT341-01 (TransGen Biotech, Beijing, China) following the protocol provided by the company. 

### 2.5. Reverse Transcription–Quantitative PCR (RT-qPCR)

The relative expression of key genes previously reported to play a major role in JA pathway was measured by RT-qPCR in the leaves of elicitor-treated (aphid-infested) and control bean plants. These JA pathway-associated genes were PHAVU_002G06700g, PHAVU_002G175500g, PHAVU_003G096400g, PHAVU_001G017800g, PHAVU_001G000800g, PHAVU_003G011600g, PHAVU_001G001300g, and PHAVU_003G111500g. Similarly, the SA pathway-associated key genes used in this study were PHAVU_006G048600, PHAVU_008G057700, PHAVU_008G272800, PHAVU_011G176100, and PHAVU_011G17200. β-actin was used as the internal control (reference gene). Primer pairs used for the RT-qPCR amplifications of these genes are given in Table S1. RT-qPCR amplifications were performed on thermocycler ABI 7500 Real-Time PCR System (Applied Biosystems, USA) using 20 µL of the reaction mixture (comprising 10 µL of 2× SYBR^®^ Premix Ex Taq (Takara, Dalian, China), 0.5 µL of each 10 μmol L^−1^ forward and reverse primers, 2 µL of 10-fold diluted cDNA template, and 7 µL of ddH_2_O). Thermal protocol included preheating at 95 °C for 30 s, followed by 40 cycles of denaturation at 95 °C for 30 s and annealing at 60 °C for 40 s and elongation at 72 °C for 60 s. There were three biological replicates for each treatment and three technical replicates for each sample. 

### 2.6. Statistical Analysis

Statistical analysis of the data was performed using Statistix Version 8.1 (Analytical Software, Tallahassee, FL, USA). The experiments were repeated three times independently and the mean values of all parameters are presented in figures along with the standard deviations. Some data (for instance, data of the fecundity of aphids) was subjected to square root transformation prior to the analysis. Significant difference among treatment factors (i.e., elicitor concentrations and temperature regimes) was determined by using one-way factorial analysis of variance (ANOVA) followed by least significant difference (LSD) test at 0.05 level of probability. The RT-qPCR expression levels were measured by using the comparative CT method (2^−∆∆*C*T^). The fold change in the treated and control samples was calculated and analyzed by Student’s *t*-test using 0.05 level of probability.

## 3. Results

### 3.1. Effect of Elicitor PeBC1 on Nymphal Development Time of Aphids

**Figure 1:**
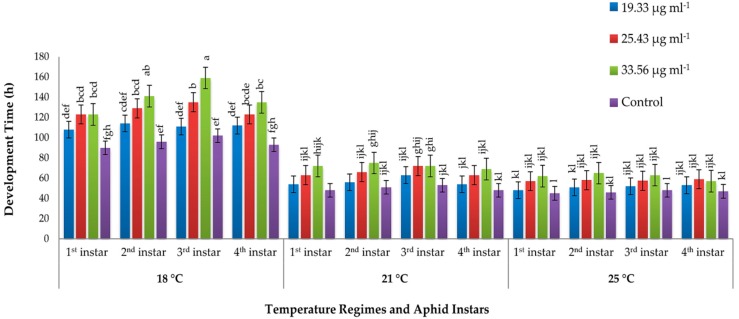
Mean nymphal development time (±SE) of green peach aphid (*Myzus persicae*) in response to the application of elicitor PeBC1 at different temperature regimes (*n* = 10). Different letters indicate significant differences among treatments (one-way factorial ANOVA; LSD at α = 0.05).

Factorial analysis of variance revealed a significant effect of the elicitor concentrations (F_3, 468_ = 17.25, *p* < 0.001), temperature regimes (F_2, 468_ = 316.79, *p* < 0.001), and their interaction (F_6, 468_ = 4.37, *p* < 0.001) on the overall nymphal development time of *M. persicae* (Table S2). Nymphal development time was prolonged along with the concentrations of the elicitor. At medium temperature (21 °C), nymphal development time was the maximum (6 days). First instar took 2 days (48 h) at control plants, while it took 2.01 (48.5 h), 2.3 (57 h), and 2.2 (54 h) days on plants treated with 33.56, 25.43, and 19.33 µg mL^−1^ of PeBC1 elicitor, respectively (Figure 1). In case of 2nd instar, it took 2.1 days (51 h) in control treatments, while at high, medium, and low concentrations of PeBC1 elicitor, it took 2.9 (75 h), 2.4 (61 h), and 2.6 days (56 h), respectively. Third instar of aphids was completed, on average, in 2.3 days (57 h) on the control plants, while with the application of high, medium, and low concentrations of elicitor, it took 2.6 (66 h), 2.8 (70 h), and 3.0 days (72 h), respectively. Similarly, 4th instar took up to 2 days (48 h) on the control plants, while with the application of high, medium, and low concentrations of PeBC1 elicitor, it took 2.2 (54 h), 2.5 (63 h), and 2.7 days (69 h), respectively (Figure 1). However, there was a differential trend recorded for nymphal development time of aphids at different temperature regimes. At low temperature (18 °C), the development time of all instars was almost double than at medium or high temperatures, most probably due to the reduced metabolic rates at low temperature. Maximum elongation was found at 3rd instar at each temperature and concentration. The effect of elicitor concentration was highly significant for the 1st instar (F_3, 108_ = 4.24, *p* = 0.01), 2nd instar (F_3, 108_ = 4.45, *p* = 0.01), 3rd instar (F_3, 108_ = 5.59, *p* = 0.001), and 4th instar (F_3, 108_ = 5.19, *p* = 0.002). Similarly, temperature also exerted a significant effect on 1st instar (F_2, 108_ = 61.12, *p* < 0.001), 2nd instar (F_2, 108_ = 68.94, *p* < 0.001), 3rd instar (F_2, 108_ = 86.10, *p* < 0.001), and 4th instar (F_2, 108_ = 56.95, *p* < 0.001) aphids. However, their interaction had a non-significant effect on the nymphal development time of aphids (Table S2).

### 3.2. Effect of Elicitor PeBC1 on the Fecundity of Aphids

**Figure 2:**
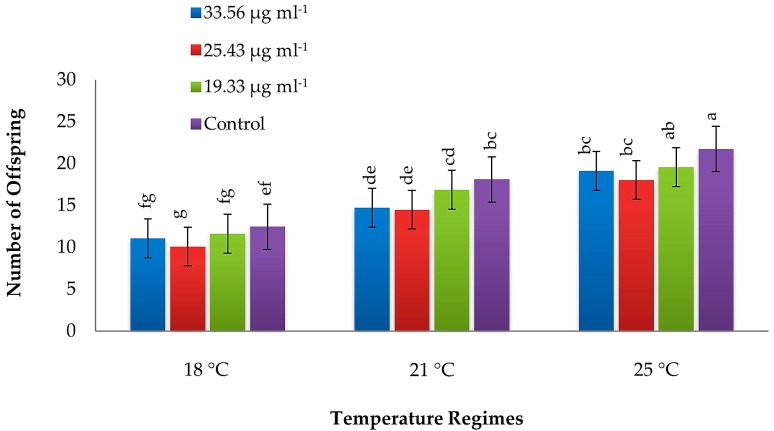
Mean fecundity (± SE) of green peach aphids (*Myzus persicae*) on common bean plants treated with different concentrations of PeBC1 elicitor at different temperature regimes (*n* = 10). Different letters indicate significant differences among treatments (one-way factorial ANOVA; LSD at α = 0.05).

The fecundity of green peach aphids was also significantly reduced by different concentrations of PeBC1 elicitor (F_3, 108_ = 24.84, *p* ≤ 0.001) and different temperature regimes (F_2, 108_ = 270.57, *p* ≤ 0.001) (Table S3). Aphids that fed on the elicitor-treated plants a produced lower number of offspring during their life cycle compared to those fed on the untreated (control) plants. Maximum fecundity was observed at high temperature (25 °C), while the fecundity was the minimum at low temperature (18 °C; Figure 2).

### 3.3. Expression Levels of JA and SA Pathway-Associated Genes in Response to PeBC1 Elicitor

**Figure 3:**
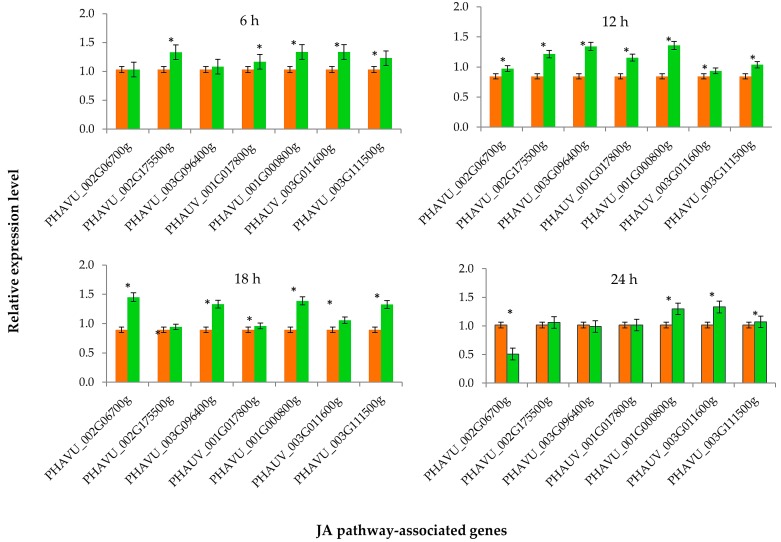
Relative expression levels of JA pathway-associated genes measured at different time intervals after the application of PeBC1 elicitor protein. Green bars indicate the result of PeBC1 elicitor treatments, while orange bars show the result of buffer-treated (control) treatments. Asterisk symbols indicate the significant difference between the treatments for each gene (Student’s *t*-test, *p* < 0.05).

**Figure 4:**
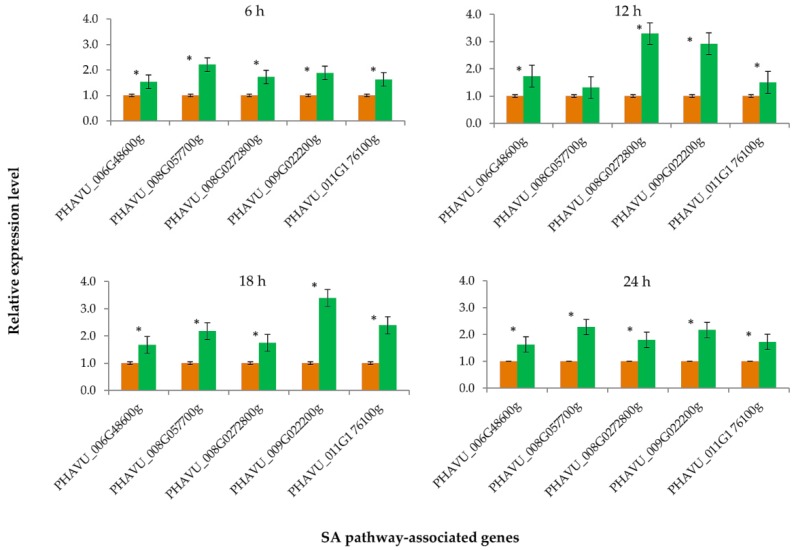
Relative expression levels of SA pathway-associated genes measured at different time intervals after the application of PeBC1 elicitor protein. Green bars indicate the result of PeBC1 elicitor treatments, while orange bars show the result of buffer-treated (control) treatments. Asterisk symbols indicate the significant difference between the treatments for each gene (Student’s *t*-test, *p* < 0.05).

After the treatment of elicitor PeBC1, the key genes of JA and SA pathways were differentially expressed in common bean plants. The expression levels of all JA pathway-associated genes were significantly different (*p* = 0.05) from each other at different time intervals (Figure 3). After 6 h of elicitor treatment, most of the JA pathway genes were slightly upregulated except one gene (PHAVU_002G06700g) that showed no significant difference from the buffer-treated (control) sample. Similarly, after 12 and 18 h of the application of elicitor PeBC1, all key genes of JA pathway were upregulated, while at 24 h, one gene (PHAVU_002G06700g) was downregulated and one gene (PHAVU_001G017800g) showed no difference from the control sample. The maximum gene expression levels were recorded at 12 and 18 h of the elicitor application. A similar trend of the expression levels was observed for all key genes associated with the SA pathway (Figure 4). The expression levels of all SA genes were upregulated at 6, 12, 18, and 24 h of the elicitor PeBC1 application and were significantly different from the buffer-treated (control) treatments. Moreover, maximum expression levels of SA pathway-associated genes were observed at 12 and 18 h of the elicitor application, as found in the case of the JA pathway (Figure 4).

## 4. Discussion

Elicitor proteins play an essential role in signaling plant defense mechanisms and emerge as important tools in biological control of insect pests. Necrotrophic, biotrophic, and other pathogenic fungi constitute an important source of microbial elicitors (MAMPs or PAMPs) [1]. This study conducted an in vitro evaluation of elicitor PeBC1, extracted from fungus *B. cinerea*, in order to determine its potential role against green peach aphids *M. persicae*. According to the results, the population of aphids on bean plants treated with PeBC1 elicitor developed significantly slower as compared to those confined on the control plants. Our results are in agreement with previous studies demonstrating the negative effects of exogenous applications of different elicitors such as JA, methyl jasmonate (MJ), and benzothiadiazole (BTH) on the population growth and other fitness traits of aphids [27,28]. Moreover, it is well known how elicitors produce induced systemic resistance against piercing-sucking insect pests [1,29]. Our results are also in accordance with a previous study [30] that demonstrated that an elicitor (methyl salicylate) played a significant role in the population reduction (up to 40%) of soybean aphids. Similarly, some studies have documented a significant decrease in the activity of herbivorous insects on tomato crops with the exogenous applications of plant defense-associated proteins such as proteinase inhibitors and polyphenol oxidase (PPO) [27,31]. 

Furthermore, analysis of variance indicates that PeBC1 treatment of plants resulted in a significant effect on the development time of each aphid instar and maximum development time was recorded for the lowest temperature tested (18 °C). These results corroborate the fact that with every degree rise in global temperature, the life cycle (development time) of an insect species would become shorter [32]. The quicker the life cycle, the higher the population of pests. Similarly, a significant impact of elicitor PeBC1 was recorded on the fecundity of aphids. It was observed that the reproductive capacity of aphids was gradually decreased after the application of the elicitor. These results are in line with the previous study [33] evidencing that the elicitor MJ induced a significantly low mean lifetime fecundity of aphids.

In agreement with previous studies, our results suggest that treatment of plants with PeBC1 elicitor has the potential to reduce population growth rates and performance of herbivorous insect pests. However, elicitors such as MJ and JA may induce the synthesis of different proteinase inhibitors in plants, as demonstrated in tomato plants [34]. Therefore, more studies are needed to shed light on the changes induced in bean plants (*P. vulgaris*) by PeBC1 application and how exactly these responses impact the fitness traits (development time and fecundity) of *M. persicae* aphids.

JA and SA pathways play major roles in inducing insect resistance response in plants. These are involved in the regulation and signaling transduction of downstream defense genes in plants, hence, producing a more effective defense response against insects [10,35,36]. The results of our study showed that aphids feeding on bean plants significantly upregulated seven key genes of the JA pathway. Both systemic and local defense responses induced by aphid feeding have been described in plant [37]. Our results revealed the local expression of JA and SA responsive genes in common bean leaves, although no systemic change in the expression levels of these genes were detected. These results are in agreement with a previous study that showed that *M. persicae* feeding induced a strong expression of SA-associated gene *PR-1*, which enhanced *PR*-proteins at feeding sites and produced local resistance in *Arabidopsis* plants [36].

Moreover, our results revealed that elicitor PeBC1 induced a strong and significant upregulation of the expression levels of all SA pathway-associated genes, while the expression levels were moderately increased for JA responsive genes. These findings corroborate that phloem-feeding herbivores, such as aphids, activate SA defense pathway-associated genes more strongly than those of the JA pathway [38,39].

## 5. Conclusions

In brief, we hypothesized a prolonged development time and reduced fecundity of green peach aphids *M. persicae* mediated by the application of PeBC1 protein elicitor. Through a series of bioassays, we demonstrated that the aphid individuals fed on bean plants treated with different concentrations of PeBC1 elicitor exhibited a prolonged nymphal development along with reduced fecundity. Moreover, molecular and functional characterization of key genes potentially involving JA and SA defense pathways showed that the application of PeBC1 elicitor exhibited a significant differential expression of these genes in common bean plants. These results suggest that such fungus-derived protein molecules may be used as novel biological pest management tools against aphids. 

## Supplementary Materials

The followings are available online at http://www.mdpi.com/2075-4450/10/2/35/s1: Table S1: Primer pairs used for RT-qPCR amplifications of the key genes involved in plant SA and JA defense pathways; Table S2: Analysis of variance (ANOVA) table for the effect of PeBC1 elicitor protein and temperature on the nymphal development time of green peach aphids (*Myzus persicae*); Table S3: Analysis of variance (ANOVA) for the effect of PeBC1 elicitor protein and temperature on the fecundity of green peach aphids (*Myzus persicae*).
