# Supplementary material for: Molecular and Functional Characterization of Elicitor PeBC1 Extracted from Botrytis cinerea Involved in the Induction of Resistance against Green Peach Aphid (Myzus persicae) in Common Beans (Phaseolus vulgaris L.)
Source: Insects. 2019 Jan 24;10(2):35. doi: 10.3390/insects10020035 (PMC6409696; doi:10.3390/insects10020035)
Supplement: Supplementary file 1 [file insects-10-00035-s001.pdf]

insects

MDPI

Supplementary Materials

Table S1. Primer pairs used for RT-qPCR amplifications of the key genes involved in plant SA and JA defense pathways

Targent Gene	Forward Sequence (5′ → 3′)	Reverse Sequence (5′ → 3′)
PHAVU_002G175500g	GAAAAGCGTGGAAAGCTACG	AGCCATGAACGATGATCTCC
PHAVU_001G017800g	GGGAGAAGCTGCTGAAACAC	CCGACCTGAATATCGAAGGA
PHAVU_003G111500g	GAATTTCCCTGCTGCTCTTG	CTGGCTTAGCCTCAGGAATG
PHAVU_001G000800g	AGCCGCATGCTGTTCTCTAT	TTTTCATGAACAGCGCTCAC
PHAVU_001G001300g	TGAAATGGCCAAGAAGGAAC	GGCGACGAGACCGTATATGT
PHAVU_002G06700g	CTGATGAGCAGCAGCAGAAG	AAACGGGCATAAACAACAGC
PHAVU_003G096400g	ACGACCATGGGTTGCTAGTC	AATGCTTCAGCTTCCTTCCA
PHAVU_003G011600g	TAGTGATGGTGCAGGAGCTG	GATGCAAAGGCCTCATTGAT
PHAVU_006G048600	CAGGATGCTTGGGATGATCT	CAAGGGCCTTTCCTACTTCC
PHAVU_008G057700	TGCTTCACATGAATGGTGGT	CAACCCAAGTCTGCCACTTT
PHAVU_008G272800	TCCTTGTTGATGCCCACATA	CAAAGAAAAAGGGGGAGAGG
PHAVU_011G176100	CCCATGCACAGTGTACCAAG	ACCAATTAACCCCCAAGGAG
PHAVU_011G17200	GCTGATTTGGGATGCTCTTC	CGTTTCCCTTGTTGAGTGGT
β-actin	GGAAAATCAGTCTCGGTTCAG	TCATACAGCAGCAAGCAC

Table S2. Analysis of variance (ANOVA) table for the effect of PeBC1 elicitor protein and temperature on the nymphal development time of green peach aphids (*Myzus persicae*).

SOV	Df	1st Instar			2nd Instar			3rd Instar			4th Instar			Df	Overall		
		MS	*F*-Value	*p*-value	*MS*	*F*-Value	*p*-value	*MS*	*F*-Value	*p*-value	*MS*	*F*-Value	*p*-value		*MS*	*F*-Value	*p*-value
Conc.	3	5.42	4.24	0.01	5.53	4.45	0.01	6.35	5.59	0.001	6.73	5.19	0.002	3	16.71	17.25	< 0.001
Temp.	2	78.09	61.12	< 0.001	85.71	68.94	< 0.001	97.78	86.10	< 0.001	73.76	56.95	< 0.001	2	306.75	316.79	< 0.001
Conc.× Temp.	6	0.41	0.32	0.92	1.51	1.21	0.31	3.97	3.50	< 0.01	1.52	1.17	0.326	6	4.23	4.37	< 0.001
Error	108	1.28			1.24			1.14			1.30			468	0.97		
Total	119													479			
Grand Mean / CV	3.22 / 35.09				3.37 / 33.12			3.56 / 29.94			3.23 / 35.18				3.26 / 30.20		

Table S3. Analysis of variance (ANOVA) for the effect of PeBC1 elicitor protein and temperature on the fecundity of green peach aphids (*Myzus persicae*).

SOV	DF	*SS*	*MS*	*F*-Value	*p*-value
Conc.	3	178.40	59.47	24.84	< 0.001
Temp.	2	1295.72	647.86	270.57	< 0.001
Conc. × Temp.	6	15.95	2.658	1.11	0.3612
Error	108	258.60	2.394		
Total	119	1748.67			
Grand Mean / CV	15.67 / 9.88				
